# Rhodolith Beds Are Major CaCO_3_ Bio-Factories in the Tropical South West Atlantic

**DOI:** 10.1371/journal.pone.0035171

**Published:** 2012-04-20

**Authors:** Gilberto M. Amado-Filho, Rodrigo L. Moura, Alex C. Bastos, Leonardo T. Salgado, Paulo Y. Sumida, Arthur Z. Guth, Ronaldo B. Francini-Filho, Guilherme H. Pereira-Filho, Douglas P. Abrantes, Poliana S. Brasileiro, Ricardo G. Bahia, Rachel N. Leal, Les Kaufman, Joanie A. Kleypas, Marcos Farina, Fabiano L. Thompson

**Affiliations:** 1 Instituto de Pesquisas Jardim Botânico do Rio de Janeiro, Rio de Janeiro, RJ, Brazil; 2 Instituto de Biologia, Universidade Federal do Rio de Janeiro, Rio de Janeiro, RJ, Brazil; 3 Departamento de Ecologia e Recursos Naturais, Universidade Federal do Espírito Santo, Vitória, ES, Brazil; 4 Instituto Oceanográfico, Universidade de São Paulo, São Paulo, SP, Brazil; 5 Centro de Ciências Aplicadas e Educação, Universidade Federal da Paraíba, Rio Tinto, PB, Brazil; 6 Departamento de Botânica, Universidade Federal Rural do Rio de Janeiro, Seropédica, RJ, Brazil; 7 Boston University Marine Program, Boston, Massachusetts, United States of America; 8 Climate and Global Dynamics, National Center for Atmospheric Research, Boulder, Colorado, United States of America; 9 Instituto de Ciências Biomédicas, Universidade Federal do Rio de Janeiro, Rio de Janeiro, RJ, Brazil; National Institute of Water & Atmospheric Research, New Zealand

## Abstract

Rhodoliths are nodules of non-geniculate coralline algae that occur in shallow waters (<150 m depth) subjected to episodic disturbance. Rhodolith beds stand with kelp beds, seagrass meadows, and coralline algal reefs as one of the world's four largest macrophyte-dominated benthic communities. Geographic distribution of rhodolith beds is discontinuous, with large concentrations off Japan, Australia and the Gulf of California, as well as in the Mediterranean, North Atlantic, eastern Caribbean and Brazil. Although there are major gaps in terms of seabed habitat mapping, the largest rhodolith beds are purported to occur off Brazil, where these communities are recorded across a wide latitudinal range (2°N - 27°S). To quantify their extent, we carried out an inter-reefal seabed habitat survey on the Abrolhos Shelf (16°50′ - 19°45′S) off eastern Brazil, and confirmed the most expansive and contiguous rhodolith bed in the world, covering about 20,900 km^2^. Distribution, extent, composition and structure of this bed were assessed with side scan sonar, remotely operated vehicles, and SCUBA. The mean rate of CaCO_3_ production was estimated from *in situ* growth assays at 1.07 kg m^−2^ yr^−1^, with a total production rate of 0.025 Gt yr^−1^, comparable to those of the world's largest biogenic CaCO_3_ deposits. These gigantic rhodolith beds, of areal extent equivalent to the Great Barrier Reef, Australia, are a critical, yet poorly understood component of the tropical South Atlantic Ocean. Based on the relatively high vulnerability of coralline algae to ocean acidification, these beds are likely to experience a profound restructuring in the coming decades.

## Introduction

Shallow water tropical benthic communities such as coral reefs are well known to be major carbonate producers in coastal areas [1]–[3], and significant progress has been made in understanding their calcium carbonate (CaCO_3_) production by mapping their global distributions [1]–[4] and by estimating mineralization rates [5]–[8]. There is growing evidence that communities dominated by crustose coralline algae (CCA) can also contribute significantly to the CaCO_3_ cycles of continental shelf ecosystems [9]–[11] due to their high rates of community CaCO_3_ production and dissolution [11]. Rhodolith beds are aggregates of nodules of non-geniculate CCA that generally occur in waters shallower than 150 m depth subjected to episodic wave or current movement, forming large expanses of hard bottom habitat [9]. Rhodolith beds stand together with kelp beds, seagrass meadows, and CCA reefs as one of the world's four largest macrophyte-dominated benthic communities [9], [12], but information on the CaCO_3_ production by rhodoliths remains scarce and biased toward temperate beds [13].

The global distribution of rhodolith beds is highly discontinuous, with larger concentrations recorded off southern Japan, western Australia and the Gulf of California, as well as in the Mediterranean and along Norway, Ireland, Scotland, northeastern Canada, the eastern Caribbean and Brazil [9]. Worldwide, there have been few attempts to map the large-scale distribution of rhodolith beds [9], but studies from the early 1970s suggest that those occurring off Brazil, between 2°N and 27°S, potentially represent one of the largest marine CaCO_3_ deposits in the world, with estimates of 2×10^11^ tons of CaCO_3_ [14]–[16]. The region known as the Abrolhos Bank (16°50′ - 19°45′S) is a ∼46,000 km^2^ expanse of the eastern Brazilian continental shelf; its inner and mid shelf encompass the largest and richest biogenic reefs in the South Atlantic [17]–[19], bearing coral assemblages dominated by Brazilian-endemic Neogene relicts of the genus *Mussismilia*. Although CCAs are recognized as the most important component of Abrolhos reefs [15]–[18], and rhodolith beds are recorded to the north and to the south of this region [20]–[21], the geographic extent and role of rhodolith beds on the Abrolhos Shelf have been largely overlooked.

Understanding the CaCO_3_ fluxes of the tropical Southwestern Atlantic hinges on accurate quantification on the extent and CaCO_3_ production of these large rhodolith beds. The aim of this work was thus to determine the size and CaCO_3_ production of the rhodolith beds within the Abrolhos Shelf. This meso-scale system may provide valuable insights on the overall role of the massive rhodolith beds of the western tropical and subtropical South American shelf. To accomplish this task, surveys using side scan sonar (SSS), remotely operated vehicles (ROVs) and mixed-gas diving, as well as *in situ* assays to estimate CaCO_3_ production were perfomed. Our analysis confirms the overwhelming importance of this CaCO_3_ bio-factory for the Southwestern Atlantic Ocean CaCO_3_ cycles.

## Results

**Figure 1:**
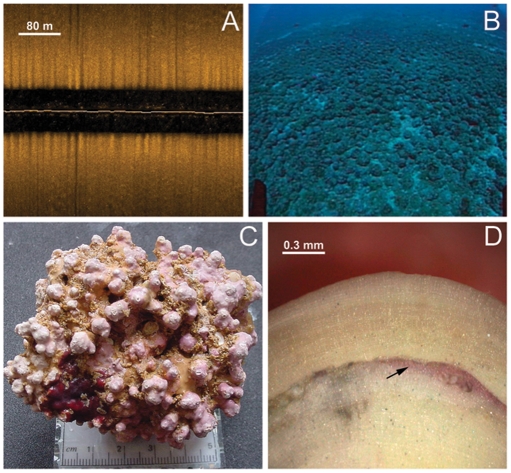
Selected aspects of rhodolith beds and individual rhodoliths. (A) SSS sonogram showing flat and highly reflective bottom typical of rhodolith beds. (B) ROV image showing the typical physiognomy of the rhodolith beds. (C) Individual rhodolith consisting primarily of *Lithothamnion crispatum* with high proportion of live tissue. (D) Superficial view of a rhodolith section observed via a stereomicroscope, showing the reddish band (arrow) corresponding to the staining performed six months earlier.

**Figure 2:**
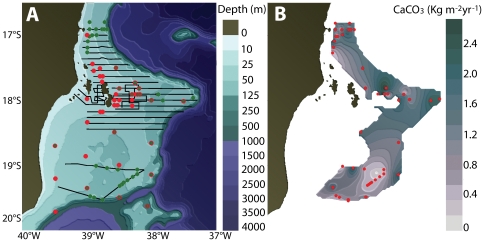
The study region off eastern South America, Abrolhos Bank. (A) Bathymetric map showing the areas surveyed with SSS (black lines), ROV ground truth sites (red dots) and mixed-gas dive collecting sites (green dots). (B) Distribution, and annual calcium carbonate production of rhodolith beds. The gray area indicates the total area occupied by the rhodolith beds, whereas the gray scale variations correspond to estimates of the annual calcium carbonate production (expressed as kg m^−2^ yr^−1^).

A representative SSS coverage was acquired for all habitat types of the mid and outer portions of Abrolhos Shelf. Flat and highly reflective bottom (Fig. 1a) predominated from depths of 20 m up to 110 m depth, indicating the presence of a low-relief hard bottom typical of rhodolith beds (Fig. 1b). This benthic feature was ground-truthed with ROVs across the entire sampled area, confirming its correspondence with rhodolith beds (100% match). In the studied area, rhodolith beds were estimated to cover a total of 20,902 km^2^ which represents at least 45% of the entire Abrolhos Shelf (Fig. 2). The mean relative cover of rhodoliths was 69.1±1.7% (± SE) while mean density was 211±20 nodules m^−2^ (Video S1). Calcareous sandy patches and patch reefs are the other primary benthic features surveyed on the mid and outer Abrolhos Shelf, while soft siliciclastic sediments, as well patch reefs and larger banks predominated on the inner shelf.

Six rhodolith-forming CCA species were identified: *Hydrolithon rupestre* (Foslie) Penrose (RB 510413, 510414), *Lithophyllum stictaeforme* (Areschoug) Hauck (RB 525142, 525148), *Mesophyllum engelhartii* (Foslie) Adey (RB 525143, 525144), *Sporolithon episporum* (Howe) Dawson (RB 525149), *Neogoniolithon brassica-florida* (Harvey) Setchell et Mason (RB 525150), and *Lithothamnion crispatum* Hauck (RB 525145, 525147). They are recognized as widespread taxa in tropical and temperate waters. *Lithothamnion crispatum* was the most abundant species within multispecific rhodoliths, as well as the most common species forming monospecific rhodoliths (Fig. 1c). The mean (± SE) rhodolith diameter was 5.9±0.2 cm and the mean percentage of live surface area was 57.3±5, indicating active growth. While rhodolith diameter appeared to increase with depth, neither rhodolith diameter nor percent live surface area was significantly correlated with depth.

The growth rates determined based on *in situ* assays were greater at 20 m depth (1.5+0.4 mm yr^−1^, n = 10) than those measured at 45 m depth (1.0+0.5 mm yr^−1^, n = 10) (t = −2.5, p<0.05).

**Figure 3:**
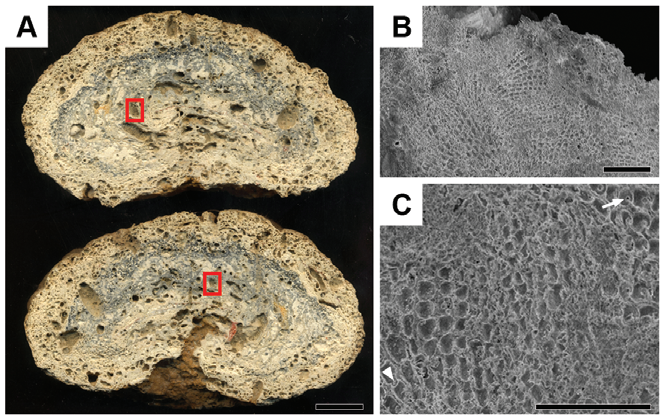
Stereomicroscopy and scanning eletron microscopy images of a isotopic dated rhodolith. (A) A section of the rhodolith made along its longest axis. The red squares indicate the regions where fragments were removed for isotopic analysis. (bar = 2 cm); (B and C) Scanning electron microscopy images showing the typical cellular organization of CCA species circumscribing the region where the fragments were collected for isotopic dating. Note in image “C" the presence of “secondary pit-connections" (arrow) and of cell fusions (arrow head) among mineralized cell walls (Bars: B = 150 um and C = 80 um).

The mean rate of net CaCO_3_ production was estimated at 1.0±0.7 kg m^−2^ yr^−1^, with a remarkable spatial variation, with values ranging between 0.3 to 2.7 kg m^−2^ yr^−1^ (Fig. 2). There were no differences in CaCO_3_ production according to depth, with large variability recorded within a given depth (e.g. 0.3–1.7 kg m^−2^ yr^−1^ at 27 m depth; Fig. 2). Rhodoliths with a mean diameter of 14 cm sampled at 65 m depth had cores composed of CCA (Fig. 3) with radiocarbon ages between 8200 and 7800 years BP.

## Discussion

Together with kelp beds, seagrass meadows, and coral reefs, rhodolith beds are one of Earth's largest macrophyte-dominated benthic communities. As expected from such a widely distributed and highly fragmented habitat type that spans from polar to tropical regions of the main ocean basins, rhodolith beds vary considerably in terms of constituent species and physiognomy (i. e. bed size and depth range, rhodolith shape and density, associated organisms as well as dynamics and disturbance regimes) [9], [13]. Furthermore, rhodoliths are widely recognized as bioengineers that provide structural complexity and relatively stable microhabitats for other species over large extensions, thus resulting in increased biodiversity and benthic primary productivity [12], [20]–[23], particularly when compared to unconsolidated flat bottom. Remarkably tough, rhodolith beds are featureless at spatial scales greater than centimeters, and many larger species of fish and invertebrates that occur on adjacent reef systems will only occasionally rove over these beds. Despite such limitations in supporting a “complete" reef assemblage, rhodolith beds likely provide migration corridors for several species when they cover large inter-reefal areas, such as in the Abrolhos Shelf. Moreover, because rhodoliths present a broad depth range within the photic zone, they are the foremost hard bottom feature in the mesophotic zone (>30 m) of the Abrolhos Shelf, where there are fewer corals species and lower coral cover (only isolated small colonies of *Mussismilia hispida* and patches of *Montastraea cavernosa* and *Siderastrea* spp.).

The world's largest rhodolith beds have been suggested to occur off Brazil [9], [13]–[16]. The results obtained here not only confirm this assertion with an unprecedented high resolution and large spatial scale assessment, but also indicate that the total area covered by rhodolith beds in the western tropical South Atlantic is larger than previously supposed. In fact, the Abrolhos Shelf encompasses the largest continuous rhodolith bed in the world, which occupies 20,902 km^2^, an area comparable to that estimated for coral reefs of the Caribbean (21,600 km^2^) and the Great Barrier Reef (20,055 km^2^) [4]. The combination of a wide and shallow tropical shelf with seasonal wave disturbance seems to provide conditions favorable to the development of such huge rhodolith beds in the Abrolhos Bank. The main reef-building corals found in the region (*Mussismilia* spp.) are restricted mostly to shallow waters and do not contribute much to carbonate deposition in larger reef blocks in the deeper parts of the Bank, where rhodoliths thrive. This contrasts with the situation observed in the reefs that occur in the inner shelf, where corals and CCA form the main framework of mushroom-shaped pinnacles that emerge from the bottom (10–25 m) and reach the surface.

Growth rates of rhodoliths from Abrolhos Shelf (1–1.5 mm yr^−1^) are similar to those reported from other studies. Extension rates of rhodolith-forming species are generally 0.5–1 mm yr^−1^ under a wide range of field and laboratory conditions (although measured rates of 0.05–2.7 mm yr^−1^ have been recorded) [24]–[29]. The calcification and accumulation rates of rhodoliths in temperate environments were summarized by Bosence and Wilson [27], who found that although the growth rates of tropical species are an order of magnitude higher than those for temperate species, the lower standing crops of the former usually result in production rates (0.06–1.0 kg CaCO_3_ m^−2^ yr^−1^) that are within the range recorded for temperate rhodoliths [27], [30], [31]. Production rates and accumulation rates for other non-geniculate corallines are also similar for both temperate and tropical sites [25], [31], and the rates recorded are not much lower than rates of coral reef formation [27].

Our age dating data indicates that some rhodoliths had cores dating to the time shortly after the Abrolhos shelf was flooded around 8000 years ago. Obviously these rhodoliths have not been growing continuously since then, and the cores represent older CCA fragments that have been re-colonized by modern rhodolith CCA. These older cores indicate, however, that rhodoliths have been present on the Abrolhos Bank since the mid-Holocene when the sea level was 60 m lower than today (Fig. 3).

Our CaCO_3_ production estimates from the Abrolhos Shelf (1.0±0.7 kg m^−2^ yr^−1^) are similar to those reported for tropical reef environments (1.3–2.7 kg m^−2^ yr^−1^; [3], [4]), and are also close to the mean global coral reef calcification rate (1.5 kg m^−2^ yr^−1^; [32]). Total CaCO_3_ production by rhodolith beds in the Abrolhos Shelf is estimated at 0.025 Gt yr^−1^, a value that is also comparable to total CaCO_3_ production by coral reefs in the Caribbean (0.04–0.08 Gt yr^−1^; [3], [4]). While net CaCO_3_ accumulation rates have not yet been measured, natural excavations (e.g. blue holes) in the rhodolith banks suggest that their thicknesses are similar to those of most modern coral reefs. The wide latitudinal span of rhodolith beds along the eastern South American coast (2°N - 27°S) and the extension of some beds, such as the one reported herein, with thousands of square kilometers, support the idea that CCA plays critical biological and physicochemical roles in the South Atlantic.

The high-magnesium calcite produced by CCA is the most soluble form of the common CaCO_3_ minerals [11], [13], and is thus highly susceptible to ocean acidification [8], [33]–[34]. Recent projections indicate that tropical CCA will stop growing by 2040 and will start to dissolve when the high-magnesium calcite saturation state is less than one [33], [34]. By the end of the century, seawater pH may decrease by as much as 0.3 pH units relative to the preindustrial [34], indicating that rhodolith beds will rapidly decline across the globe, at faster rates than those expected for coral reefs. The slow growth rate and long life-span of CCA [9], [13] indicate a low resilience to such major disturbances. The unprecedented rate of change in seawater chemistry, which is over 1,000 times faster than that of the last 420,000 years, makes the adaptation of CCA to such environmental changes unlikely [35]. The decline or disappearance of CCA in the near future could have dramatic biological and physico-chemical consequences on a global scale [13], [33]–[36], and can be even more acute in the eastern tropical shelf of South America, where rhodolith beds occupy vast areas. Increasing partial pressure of CO_2_ (pCO_2_) is projected to reduce marine calcification 40% by year 2100 and 90% by 2300 [32]. Consequently, changes in CCA carbonate production and the dissolution induced by elevated pCO_2_ and temperature will have major implications for carbon dynamics, from the carbonate chemistry of the water column to the ability of the oceans to uptake atmospheric CO_2_ [32]. Ocean acidification will cause carbonate dissolution to increase within rhodolith beds, thus causing major habitat loss for several species. Besides the high diversity of associated organisms, rhodolith beds have been shown as the primary habitat for small-range (endemic) species [21], [37], [38].

The importance of the Southwestern Atlantic rhodolith beds, particularly those of the Abrolhos Bank, has been largely underestimated. The total annual CaCO_3_ production by these beds is comparable to that of the largest biogenic CaCO_3_ deposits in the world. The gigantic CaCO_3_ bio-factory reported herein from the Abrolhos Shelf alone accounts for approximately 5% of the world's total carbonate banks. Although the main stressor (ocean acidification) is not manageable at regional scales, a broad array of threats to the Abrolhos' rhodolith beds can be managed and deserves immediate attention. These include sedimentation from land-based sources and large scale dredging and mining. Although the accumulation rate of rhodoliths may appear rapid on a geological time scale (∼1 m 10^3^ yr), it is very slow on a human time scale (∼1 mm yr^−1^). Therefore, rhodoliths should be regarded as non-renewable resources and valuable providers of critical ecosystem services.

## Materials and Methods

Benthic surveys were carried out on the Abrolhos Shelf (16°50′W, 19°45′S), off the eastern tropical coast of Brazil (Fig. 2) between 2007 and 2009. A combination of SSS sampling and video imaging with ROVs was used to determine and validate sea-bottom features, respectively. Mixed-gas technical diving was performed to collect rhodolith samples and to carry out *in situ* assays on growth rate of rhodolith-forming CCA.

Side Scan Sonar surveys included 20 cross-shelf transects between 10 and 110 m depths (Fig. 2). An Edgetech 4100 system with a 272TD towfish was operated at 100 kHz in 200 and 400 m swaths. Acoustic data were processed using SonarWis Map4 software; geo-referenced mosaics were exported as GeoTiff images with 1 m/pixel resolution into ArcGIS 9.2, while morphological attributes such as area and depth were treated as shapes.

A Seabotix® LBV 150S2 ROV equipped with a color video camera and a pair of scaling lasers (5 cm apart) was used to validate sea-bottom features recorded with SSS. Footage was recorded for at least 40 minutes in each deployment, covering the main benthic features at each site. A total of 52 sites identified by SSS as rhodolith bed (Fig. 2) were validated with ROV sampling. Footage was transformed into one-frame-per-second still images, from which 25 randomly selected frames were used to determine the coverage, density (rhodoliths m^−2^), and dimensions of rhodoliths at all 52 sites.

Sixty rhodoliths were collected at each site. Immediately after collection, the top surface of each specimen was photographed to record the color of CCA thallus, which was used to estimate the percent surface area with live tissue (i.e. percent live surface area). Photographs were analyzed using Coral Point Count with Excel Extensions software [39], with 50 sampling points randomly positioned over each rhodolith image. Percent live surface area was determined as the percentage of total points over living algae thallus (shades of red on the image). The largest, intermediate, and smallest diameters were measured to the nearest millimeter with a measuring tape.

Identification of CCA species composing the external surface of rhodoliths was conducted using pieces of fertile material submitted to both light and scanning electron microscopy (SEM). Samples were decalcified in 10% nitric acid, dehydrated in ethanol, embedded in resin, and sectioned (5–10 µm thick) for observation under a light microscope. Specimens examined with SEM were air dried and mounted on aluminum stubs with a double-sided conductive tape. Stubs were sputter coated with gold and investigated with a Zeiss EVO 40 SEM at 15 kV. The species were identified according to recent studies of CCA taxonomy [23], [37], [40]–[42]. Voucher specimens were included in the herbarium of Rio de Janeiro Botanical Garden (RB).

Calcification rates of rhodoliths were determined *in situ* in May 2009, rhodoliths from the central area of the Abrolhos Shelf were selected from 20 and 45 m depths (ten rhodoliths from each depth strata). Each rhodolith was tagged with thin plastic tape and stained in an aerated 0.025% (w/v) alizarin red seawater solution for 24 h, in order to create a detectable band (time line) on the epithelial margin layer of the CCA crust. Rhodoliths were replaced at each corresponding site in a 1×1×0.5 m open-topped enclosure among other unstained individuals. After six months rhodoliths were recovered and sectioned. The distance from the stained region to the newly grown epithelial margin layer was measured (Fig. 1d). At least 20 measurements were taken for each rhodolith, and a mean rhodolith thickness was obtained for each depth (mm yr^−1^).

The mass of CCA accumulated (g m^−2^ yr^−1^) was obtained from mean density, dimension, and percent live surface data, together with the estimates of annual growth of rhodolith-forming CCA. It was taken into account that 99% of CCA mass obtained after calcinations process (72 h at 400°C) correspond to CaCO_3_. Calculations were performed as follows.

Rhodoliths have an ellipsoid form, with volumes (*V*) determined by the equation:
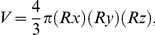
where *Rx*, *Ry*, and *Rz* are the shortest, intermediate, and largest radii, respectively.

Increase in rhodolith volume after one year (*V′*) was obtained by subtracting initial from final volumes with the following equation:
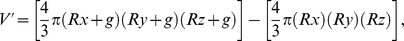
where *g* is rhodolith extension in cm after one year. Values obtained by this method were previously validated by comparing with volume estimates from liquid volume displacement, with a strongly positive correlation between the two methods (Pearson correlation coefficient; R = 0.98). Based on *in situ* assays, the increase in rhodolith thickness was estimated at 1.5 mm yr^−1^ down to 40 m depth and 1 mm yr^−1^ between 40 m and 110 m.

Density (d) is defined as mass/volume, with CCA density estimated at ∼1.56 g cm^−3^ [43]. Increase in CaCO_3_ mass on a rhodolith bed was estimated for a one-year period (M) with the following equation:
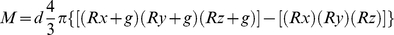
CaCO_3_ production rate (CaCO_3_pr, expressed in g m^−2^ yr^−1^) was obtained with the following equation:
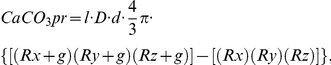
where l is rhodolith mean % live surface (ranging between 0 and 1) and D is mean rhodolith abundance (ind m^−2^). The total CaCO_3_ production by rhodoliths was calculated using a 3-D integral of production rates measured for the Abrolhos Bank.

Radiocarbon age (^14^C) was determined in samples from the core of three typical rhodoliths collected at 65 m depth. After sawing the rhodolith into two halves, a square piece of 2 mm core of rhodolith was removed and observed by scanning electron microscopy (Fig. 3) After confirming that the fragment was composed of CCA, the it was sent to Beta Analytic Inc. (Miami, Florida) for analyses by Accelerator Mass Spectrometry (AMS). Results were derived from reduction of sample carbon after acid etch pretreatment to graphite (100%C) with subsequent detection in AMS. Dates are reported as RCYBP (readiocarbon years before present, “present" = AD 1950).

## Supporting Information

Video S1 **Video obtained by ROV showing three areas within the Abrolhos Bank that are typically dominated by rhodolith beds: stations 35 (80 m depth), 48 (67 m depth), and 59 (31 m depth).** Note that most of the substrate was covered by live CCA (shades of red) forming rhodoliths.
(MOV)
